# Real-Time Monitoring of SARS-CoV-2 Variants in Oklahoma Wastewater through Allele-Specific RT-qPCR

**DOI:** 10.3390/microorganisms12102001

**Published:** 2024-09-30

**Authors:** Kristen Shelton, Gargi N. Deshpande, Gilson J. Sanchez, Jason R. Vogel, A. Caitlin Miller, Gabriel Florea, Erin R. Jeffries, Kara B. De Leόn, Bradley Stevenson, Katrin Gaardbo Kuhn

**Affiliations:** 1 School of Civil Engineering and Environmental Science, University of Oklahoma, Norman, OK 73071, USA; kristen.k.shelton-1@ou.edu (K.S.); sanchez.gj@ou.edu (G.J.S.); jason.vogel@ou.edu (J.R.V.); caitlin.miller@ou.edu (A.C.M.); 2 Department of Biostatistics and Epidemiology, Hudson College of Public Health, University of Oklahoma Health Sciences Center, Oklahoma City, OK 73104, USA; gargi-deshpande@ouhsc.edu; 3 Oklahoma Medical Research Foundation, Oklahoma City, OK 73104, USA; gabriel-florea@omrf.org; 4 School of Biological Sciences, University of Oklahoma, Norman, OK 73019, USA; erin.jeffries@ou.edu (E.R.J.); deleonkb@ou.edu (K.B.D.L.); bradley.stevenson@ou.edu (B.S.); 5 Earth and Planetary Science, Northwestern University, Evanston, IL 60208, USA

**Keywords:** wastewater surveillance, SARS-CoV-2, variant, sequencing, Omicron, RT-qPCR, early warning

## Abstract

During the COVID-19 pandemic, wastewater surveillance was used to monitor community transmission of SARS-CoV-2. As new genetic variants emerged, the need for timely identification of these variants in wastewater became an important focus. In response to increased reports of Omicron transmission across the United States, the Oklahoma Wastewater Surveillance team utilized allele-specific RT-qPCR assays to detect and differentiate variants, such as Omicron, from other variants found in wastewater in Oklahoma. The PCR assays showed presence of the Omicron variant in Oklahoma on average two weeks before official reports, which was confirmed through genomic sequencing of selected wastewater samples. Through continued surveillance from November 2021 to January 2022, we also demonstrated the transition from prevalence of the Delta variant to prevalence of the Omicron variant in local communities. We further assessed how this transition correlated with certain demographic factors characterizing each community. Our results highlight RT-qPCR assays as a rapid, simple, and cost-effective method for monitoring the community spread of SARS-CoV-2 genetic variants in wastewater. Additionally, they demonstrate that specific demographic factors such as ethnic composition and household income can correlate with the timing of SARS-CoV-2 variant introduction and spread.

## 1. Introduction

Throughout the COVID-19 pandemic, regular surveillance of newly introduced SARS-CoV-2 variants allowed for better understanding of disease transmission patterns and ultimately better preparedness for possible outbreaks and surges [1]. The emergence of new variants stems from SARS-CoV-2 replication within its hosts over successive cycles, leading to the accumulation of numerous mutations, to include, but not limited to, the receptor-binding domain [2,3]. Collectively, these mutations in the genetic makeup of SARS-CoV-2 provide fitness advantages such as improved transmission, infectivity, different tropism, modulated virulence, and evasion of the immune response triggered by vaccination or prior infection [4]. When evidence of B.1.1.529 SARS-CoV-2 Omicron variant transmission emerged in November 2021, Omicron was highlighted as a variant of concern by the World Health Organization (WHO) [5]. When initial reports suggested that the Omicron variant was associated with increased transmissibility and potential immune evasion, an expansive international response of increased prevention, control, and surveillance measures was triggered [6], including focused attempts to confirm the presence of the variant in wastewater.

At the peak of the pandemic, wastewater surveillance proved to be a highly effective tool for early warning of increased community transmission of SARS-CoV-2, allowing for timely public health responses in areas with increasing concentrations of the virus in wastewater [7,8,9]. The relevance of this surveillance method to detecting genetic variants of SARS-CoV-2 was also highlighted during the emergence of different variants including the Delta (B.1.617.2) variant in mid-2021, as well as subsequent variants in later periods [10,11,12]. In general, variant-specific wastewater surveillance provided data for prevention and control approaches and important knowledge for understanding the transmission dynamics of each variant. For the Omicron variant, its specific genetic characteristic comprises the ‘S-gene target failure’ (SGTF)—a mutation in the S-gene which resulted in a deletion of amino acids at position 69 and 70 [13,14]. The SGTF is characteristic for both the Omicron and the originally circulating Alpha (B.1.1.7) variants; however, it was not found in the previously abundant Delta variant. Therefore, the detection of the SGTF in samples at this time was highly suggestive of the presence of Omicron. 

Next-generation sequencing (NGS) has traditionally been used for genetic sequencing to confirm genotypes and to assess genetic mutations of bacterial or viral genomes found in human clinical samples; it has also proven to be reliable for similar analysis of wastewater samples [15]. However, this analytical method is time-consuming, often expensive, and requires expert knowledge in computational biology. Furthermore, this method may not result in the detection of variants found in low abundance and can yield inconclusive, low-quality results on samples extracted from wastewater [16]. As an alternative to genetic sequencing, some laboratories have relied on Real-Time Quantitative Reverse Transcription Polymerase Chain Reaction (RT-qPCR) assays, which generate results in real time and can indicate the presence of variant-specific mutations in gene targets of samples isolated from either human samples or wastewater [17,18,19]. 

Both NGS and RT-qPCR were successfully used to identify the SARS-CoV-2 Omicron variant in wastewater collected from several cities across the US, Europe, Asia, and Africa [20,21,22,23,24]. 

In this paper, we report the initial detection of Omicron in wastewater from several locations throughout Oklahoma and demonstrate how targeted RT-qPCR can be used routinely for assessing real-time spread and dynamics of the variant in different communities. 

## 2. Materials and Methods

### 2.1. Wastewater Sampling and Sample Preparation

From 1 November 2021 to 31 January 2022, we collected wastewater samples twice per week from wastewater treatment plants (WWTPs) in the City of Anadarko (one), the City of Tulsa (three), and Oklahoma City (four), as well as 13 sewersheds in the city of Oklahoma City. The WWTP samples were collected as either time-weighted composite or flow-weighted composite by employees at each respective WWTP and transported appropriately to our lab for processing. All sewershed samples were collected in a similar manner as previously described by Kuhn et al. [7]. Modifications to sample collection were limited to collection type (either time-weighted composite sample or flow-weighted composite sample).

Three technical replicates, each with a volume of 32 mL, were prepared for nucleic acid extraction from each wastewater sample. Each sample was passed through a 70 µm nylon mesh cell strainer and amended with Bovine Coronavirus (BCoV) as an internal control by adding 100 uL of a 1:100 dilution of the reconstituted Bovine Rota-Coronavirus Vaccine (Calf Guard^®^, Zoetis, Parsippany, NJ, USA). Virus particles were concentrated via a PEG precipitation modified after Wu et al. [25], in which 8 mL of a solution containing 125 μM PEG8000 and 2M NaCl2 was added to each sample and vortexed to mix. After overnight incubation at 4 °C (12–16 h), virus particles were collected via centrifugation (14,500× *g*, 4 °C, 45 min). Supernatant was decanted and pelleted solids were used for total nucleic acid extractions following a protocol modified from the Bio On Magnetic Beads (BOMB) platform [26], described in Kuhn et al. [7].

### 2.2. Molecular Analysis 

Nucleic acids from extractions were quantified using the Reverse Transcriptase Quantitative Polymerase Chain Reaction (RT-qPCR) with primers and probes (nCOV_N1; Integrated DNA Technologies, Inc. Coralville, IA, USA) used in the Centers for Disease Control and Prevention (CDC) 2019-nCoV Real-Time RT-PCR Diagnostic Panel instructions for use under CDC’s Emergency Use Authorization [27] as previously described by Kuhn et al. [7]. 

Known variants of SARS-CoV-2 were identified with the TaqMan SARS-CoV-2 Mutation Research Panel (ThermoFisher, Waltham MA USA) [28], which targeted two different alleles: an S-gene target containing a 69–70del S-gene mutation (i.e., SGTF) and a ‘wild-type’ (WT) allele that does not contain said deletion. Allele-specific RT-qPCR assays allow for discrimination of two variants, using specific primers and probes that target single-nucleotide polymorphisms of the target gene. Detection of targets containing the 69–70del S-gene mutation were identified as the Omicron variant and WT alleles were presumed to be the Delta variant. The mutation research panel was designed according to the protocol (Revision C.0) developed by the manufacturer [28] but modified by replacing the TaqPath mastermix CG with the TaqPath™ 1-Step Multiplex Master Mix No ROX (Applied Biosystems, Waltham, MA, USA). Primers and probes used in this assay are proprietary [28]. The reaction mix contained 2.5 µL TaqPath™ 1-Step Multiplex Master Mix No ROX, 0.25 µL TaqMan™ SARS-CoV-2 Mutation Panel Assay (40×) (S.delH69V70), and 4.75 µL nuclease-free treated water was added to each well of a 0.1 mL, 96-well plate. Each reaction contained 2.5 μL wastewater nucleic acid extract diluted 1:4; synthetic RNA (positive control) or nuclease-free treated water (negative control) was added for a total volume of 10 µL. The following thermocycling parameters were used for PCR amplification: 60 °C for 30 s, 50 °C for 10 min, and 95 °C for 2 min; then 45 cycles of 95 °C for 30 s and 60 °C for 30 s; and a final elongation step of 60 °C for 30 s. The quantity of template molecules (SARS-CoV-2 viral genome equivalents) in each reaction was estimated using a standard curve of triplicate reactions containing 10^1^, 10^2^, 10^3^, 10^4^, and 10^5^ copies of the Twist Alpha control 14 and the Twist Delta control 23 [29]. 

### 2.3. SARS-CoV-2 Genome Alignment and Variant Detection

To confirm the presence of the Omicron variant in the wastewater samples analyzed in this study, we performed high-throughput sequencing across the entire genome of SARS-CoV-2. We concentrated specifically on the period between December 2021 and January 2022, as it marked the transition from the Delta variant to Omicron. For this purpose, we extracted total nucleic acids from eight wastewater samples collected in Oklahoma City and submitted them for paired-end (150 bp) high-throughput sequencing. cDNA was synthesized with LunaScript^®^ RT SuperMix Kit (NEB) per manufacturer’s protocol. Samples were then prepared for sequencing using xGen™ SARS-CoV-2 Amplicon Panel with xGen Amplicon UDI Primer Plate 1 (IDT) per manufacturer’s protocol. Samples were checked for quality and sequenced at the Oklahoma Medical Research Foundation using Illumina NextSeq 500 Mid-output 300 cycle runs with PE150 reads. Paired-end 150 bp FASTQ files were processed using the C-WAP bioinformatics pipeline (https://www.github.com/CFSAN-Biostatistics/C-WAP, accessed on 15 May 2023) [30], referencing the severe acute respiratory syndrome coronavirus 2 (SARS-CoV-2) isolate Wuhan-Hu-1 genome (Genbank ID: NC_045512.2). Variant prevalence was calculated using Freyja version 1.4.2 (https://github.com/andersen-lab/Freyja, accessed on 15 May 2023) [31]. 

### 2.4. Epidemiological Data and Analyses

We calculated 10-day moving averages for SARS-CoV-2, Omicron, and Delta concentrations at sewersheds and WWTPs. Concentrations of Omicron and Delta variants at sewersheds and WWTPs were used to calculate the relative proportion of Omicron over time. For each sewershed, we obtained estimates on population sizes, population density, and demographic variables (e.g., ethnic composition, proportion of population aged 65 years or older, and median income) using census-traction portion estimates clipped to sewershed/facility polygon boundaries [7]. The average number of days for introduction of and dominance of Omicron was calculated and compared between explanatory variables (e.g., location type, demographic characteristics) with descriptive statistics and *t*-tests. We analyzed days to introduction and dominance of Omicron and concentrations of Omicron in relation to demographic information using generalized linear univariate and multivariate modeling. All analyses were adjusted for the effect of population size and population density. All statistical analyses were performed in STATA 17 [32].

## 3. Results 

Between 1 November 2021 and 31 January 2022, we collected and analyzed 466 wastewater samples from 13 neighborhood sewersheds and 9 wastewater WWTPs for concentrations of total SARS-CoV-2, sequences that are wild-type at amino acids H69 and V70 in the virus S gene (presumed to be the Delta variant based on circulating variants at that time), and sequences conferring a deletion of amino acids H69 and V70 in the virus S gene (SGTF variant; Omicron). 

### 3.1. SARS-CoV-2 Variant Detection

**Figure 1:**
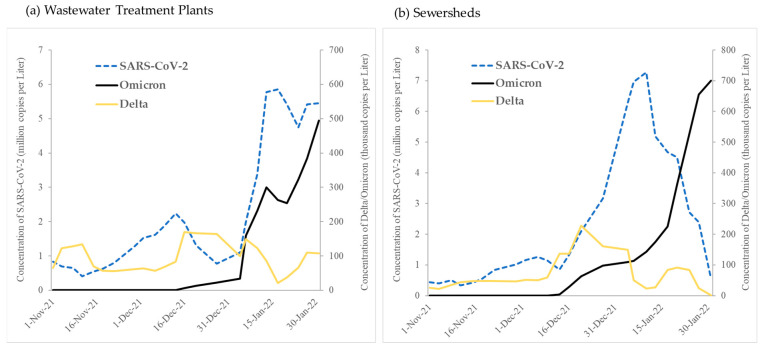
Average concentrations (10-day moving averages) of SARS-CoV, Delta and Omicron variants in (**a**) wastewater treatment plants and (**b**) sewersheds in Oklahoma, November 2021–January 2022.

**Figure 2:**
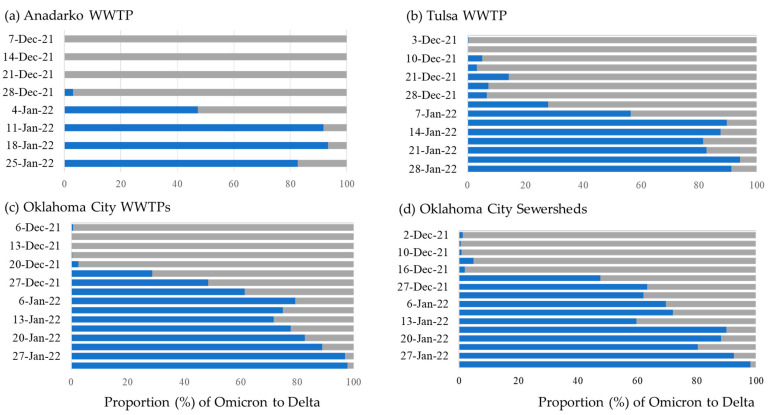
Average proportion (%) of Omicron 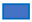 and Delta 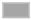 variants in Oklahoma wastewater treatment plants (WWTP) and sewersheds, Oklahoma 2021–2022.

Allele-specific RT-qPCR analysis resulted in the first detection of SGTF in early December 2021. Subsequently, there was a rapid increase in SGTF detection through January 2022 in both WWTPs and sewersheds, while Delta detection decreased beginning January 2022 (Figure 1). We identified a faint but positive SGTF detection in the Oklahoma City WWTP and associated sewershed in early December 2022 (Figure 2), which was confirmed to be the Omicron variant through NGS. The presence of SGTF (Omicron) increased, becoming the overwhelmingly abundant genotype found in both Oklahoma City and Tulsa WWTPs and sewersheds between December 2021 and January 2022 (Figure 2). 

**Figure 3:**
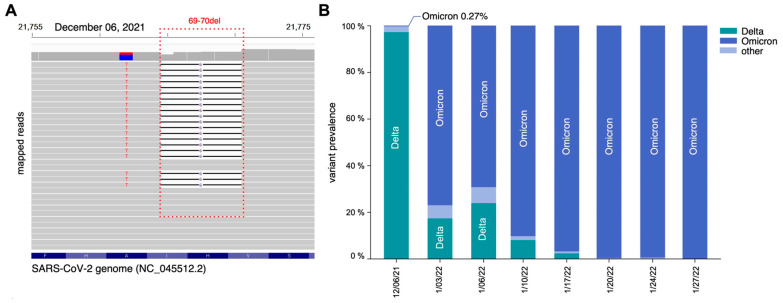
Detection of the Omicron strain through variant-specific genome deletion analysis. (**A**) Genome browser snapshot depicting the 69–70del mutation within the Spike protein coding region of SARS-CoV-2 and showing mixed presence and absence of the Omicron-specific 69–70del mutation (dotted red rectangle). (**B**) Regional prevalence estimates for daily occurrences of SARS-CoV-2 variants illustrates circulating variants at the regional level, with the "other" category consolidating lineages below 1%, excluding Delta and Omicron.

**Figure 4:**
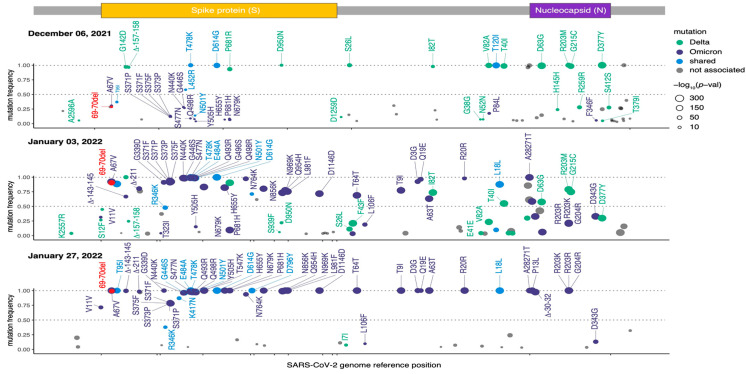
Representative genomic region of SARS-CoV-2 highlighting mutations associated with variants: Delta (green), Omicron (purple), shared by Delta and Omicron (blue), and unassigned (grey). The Omicron-specific deletion at 60-71del is indicated in red, with mutation frequency shown on the Y-axis.

For the sequencing, all sequenced samples had an average of 1.52 × 10^5^ mapped paired-end reads and a genome coverage exceeding 80% with at least 10 reads per nucleotide (10×). Visual inspection of the mapped reads from the 6 December 2021 sample confirmed the presence of the six-nucleotide deletion corresponding to histidine-69 (S:H69) and valine-70 (S:V70) (Figure 3A, red dotted line). Analysis of variant prevalence across the eight samples revealed the Omicron variant at a frequency of 0.27% on 6 December 2021, which had completely taken over by 20 January 2022 (>99%) (Figure 3B). Further examination of sequences from the 6 December 2021 sample revealed additional Omicron-specific mutations within the spike coding region, including A69V, S375F, Q498R, E484A, Y505H, and H655Y (Figure 4, purple points). Similarly, samples from January 2022 showed a marked increase in mutations specific to the Omicron variant, particularly within the spike (S) and nucleocapsid (N) genes (Figure 4, purple points in middle and bottom panel). Additional affirmation that our PCR-based assay detected the Omicron variant was provided by the absence of Alpha-specific mutations, such as A570D, T716I, and S982A (Figure 4, purple points). 

Collectively, these findings strongly support the suitability of our PCR-based assay to monitor the emergence of Omicron in our particular wastewater samples by targeting the two successive amino acid deletions (S:H69 and S:V70).

### 3.2. Timing of Omicron Introduction

Our first detection of the SARS-CoV-2 Omicron variant was an average of 48 days after the start of the study period (ranging from 11 to 73 days, Table 1). The variant was first detected in an Oklahoma City wastewater treatment facility on 11 November 2021, and in an Oklahoma City sewershed on 2 December 2021 (Table 1). For the Tulsa and Anadarko WWTPs, we first detected the Omicron variant on 10 December and 28 December 2021, respectively (Table 1). 

Generally, concentrations of SARS-CoV-2 and the proportion of Omicron detected in wastewater samples increased during the study period, with SARS-CoV-2 peaking and then decreasing from 10 January 2022, while the proportion of Omicron continued to increase (Figure 2, Table 1). 

### 3.3. Timing of Omicron Dominance

Throughout the study period, the Omicron vs. Delta proportion increased markedly in all WWTPs and sewersheds (Figure 1), reaching 100% in several locations at the end of January 2022. For all locations, it lasted an average of 63 days from 1 November 2021, until the proportion of Omicron exceeded 50% (Table 1). This progression was significantly longer in sewersheds compared to treatment plants (t = 4.4, df = 423, *p* < 0.001). We also observed a reverse relationship between Omicron introduction and the time until the proportion of Omicron exceeded 50% where locations with a longer introduction time experienced more rapid dominance of Omicron and vice versa (z = −6.1, 95%CI: −0.3–−0.1, *p* < 0.05). 

### 3.4. Demography and SARS-CoV-2 Variants

**Figure 5:**
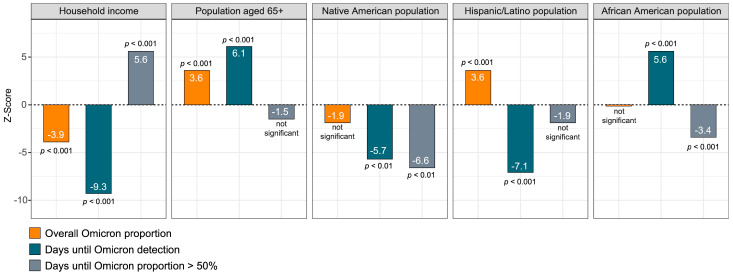
Association between Omicron wastewater indicators and demographic factors, Oklahoma 2021–2022.

We explored univariate relationships between demographic characteristics and the indicators of Omicron and Delta in sewersheds (Figure 5). The ethnic composition of monitoring locations significantly impacted when Omicron was first detected. The detection of Omicron occurred later in areas with larger African American and American Indian populations, smaller Hispanic/Latnio populations, lower household incomes, and more persons aged 65+. Conversely, these same areas also had a more rapid progression to Omicron dominance and some evidence of higher Omicron concentrations in general (Figure 5). 

Combining the wastewater indicators and demographic variables in multivariate models showed that later detection of Omicron was observed in areas with larger African American and American Indian populations and more persons aged 65+ (z = 17.6, 95%CI:1.6–2.0, *p* < 0.001). On the other hand, the number of days before the proportion of Omicron exceeded 50% was lower in those same areas and higher in areas with a high household income (z = 64.8, 95%CI:4.3–4.5, <0.001). 

## 4. Discussion

In this paper, we use evidence from routine wastewater surveillance of SARS-CoV-2 in several locations across the State of Oklahoma to highlight the dynamic transition of a SARS-CoV-2 variant that was newly introduced in November 2021. The strength and sustainability of our results are founded in the use of sensitive PCR techniques and their reliability is confirmed by next-generation sequencing, highlighting that the PCR assays provide an accurate, cost-effective, real-time alternative to the more expensive and time-consuming sequencing methods

The SARS-CoV-2 Omicron variant was first reported in South Africa and immediately noted as a variant of concern by the WHO on 24 November 2021 [33]. Following this, evidence emerged that the variant was present in human samples collected in the Netherlands on 19 November [34] and wastewater samples collected in New York on November 21 [20]. In this paper, we present results that demonstrate the presence of Omicron in Oklahoma wastewater as early as 11 and 19 November, lending support to other observations of the variant outside South Africa before 24 November. This also provides evidence to back up the hypothesis that the Omicron variant was circulating worldwide before the first official notifications.

In addition to the early detection of a SARS-CoV-2 variant of concern in Oklahoma, our results also highlight the significance of and potential for using RT-qPCR assays as a simple, rapid, and cost-effective method of detecting the emergence and community transmission of novel SARS-CoV-2 variants. Because the PCR assay offers a realistic alternative for variant tracking, this finding is especially relevant for national surveillance institutes and laboratories who lack timely access to traditional sequencing equipment and expertise. Considering the longer processing times for genomic sequencing, our method also has the distinct advantage of providing real-time ‘early warning’ for healthcare providers needing updated information for control, treatment, and prevention action. 

Our results indicate that Omicron was introduced in Oklahoma neighborhoods from mid-November to early/late December 2021 and rapidly became dominant over Delta, corresponding to what was reported from other wastewater surveillance efforts across the US [20,35,36]. We show that, until mid-late January 2022, increases in overall concentrations of SARS-CoV-2 in wastewater seemed largely driven by an increase in Omicron’s proportion. This lends further support to the observed high infectiousness and rapid spread of the variant in most areas across the world. By combining wastewater indicators with demographic data, we also demonstrate how the introduction of Omicron and its transition towards becoming the dominant variant was influenced by the ethnic composition of the area covered by the treatment plant or sewershed. Omicron was introduced later in African American and American Indian populations and among persons aged 65 or older; however, once present, it rapidly became dominant. Interestingly, areas with higher household incomes experienced a more rapid introduction but slower spread of Omicron. The Delta variant disproportionately affected specific ethnic groups and lower-income populations [37,38], and these may have still experienced some cross-protective immunity from a Delta infection, causing the introduction of Omicron to be delayed. Following established transmission, the dominance of Omicron would be rapid because of its high infectiousness. In ethnically diverse and low-income communities, the time to Omicron dominance was most likely further shortened because of reduced healthcare access and reduced access to—and use of—personal protective equipment [39,40,41]. 

Our study presents relevant insights into the detection of a novel SARS-CoV-2 in wastewater and evidence for how this variant spread and became dominant across diverse population groups in Oklahoma. However, the results need to be interpreted with consideration of potential limitations. Firstly, primers may selectively amplify a single variant from a diverse pool, which could result in an unbalanced representation of the variants present. Secondly, our approach operates under the assumption that the prevalence of both Omicron and Delta variants mirrors the national trends observed at the time of the analysis. Recent evidence nonetheless suggests a significant deviation from this assumption, particularly in Oklahoma [42]. Further, reports of ‘Long COVID’ are comparatively high in Oklahoma [42], highlighting a continued presence and possible overabundance of older variants, including the Alpha variant, which also presents the 69–70del mutation [43,44]. This finding implies that the viral landscape in Oklahoma may not align with national trends, potentially due to localized factors or the persistence of earlier variant strains. Finally, due to data sparsity, we did not investigate and account for interactions between demographic variables such as ethnic composition and household income. Such interactions, if present, could alter the magnitude of effects observed in the multivariate models, but are unlikely to change the direction of the outcome. 

Wastewater surveillance has proven to be a highly effective and useful tool for monitoring trends in SARS-CoV-2, including measuring community transmission dynamics of genetic variants. Our results from the State of Oklahoma confirm this with an added novel aspect of using allele-specific RT-qPCR as a timelier alternative to traditional genomic sequencing. To our knowledge, we also present the first results to show how the introduction and spread of Omicron differed significantly between areas depending on ethnic and socioeconomic composition. This evidence can ultimately form a crucial foundation for healthcare providers and decision-makers with respect to planning and implementing future variant-focused control and prevention measures. 

## Figures and Tables

**Table 1:** Characteristics of SARS-CoV-2 Omicron variant detection in wastewater treatment plant (WWTP) and sewersheds from 1 November 2021 to 31 January 2022, Oklahoma.

Location	Average Number of Days until First Detection (min, max)	Date of First Detection (Copies/Liter)	Date of Peak Concentrations in Wastewater (Copies/Liter)	Average Number of Days until Proportion >50% (min, max)
Anadarko WWTP	58 (n.a)	28 Dec 2021 (4397)	18 Jan 2022 (383,986)	71 (n.a)
Tulsa WWTP	48 (40, 51)	10 Dec 2021 (6517)	21 Jan 2022 (950,557)	64 (64, 64)
Oklahoma City WWTPs	40 (11, 50)	11 Nov 2021 (5747)	2 Jan 2022 (1,523,688)	62 (56, 66)
Oklahoma City sewersheds	50 (32, 65)	2 Dec 2021 (4881)	24 Jan 2022 (811,981)	62 (42, 73)

## Data Availability

Measures of raw wastewater concentrations by location and date, including all corresponding positive and negative controls, as well as concentrations of Omicron and wild-type variants by location and date, are available upon request to the corresponding author.
